# Supplementary material for: The causal role of circulating inflammatory markers in osteoporosis: a bidirectional Mendelian randomized study
Source: Front Immunol. 2024 Jul 18;15:1412298. doi: 10.3389/fimmu.2024.1412298 (PMC11291241; doi:10.3389/fimmu.2024.1412298)
Supplement: Supplementary file 1 [file DataSheet_1.zip › supplementary material/OSTEOPOROSIS-CIM/GCST90274827.tsv.gz_forest.pdf]

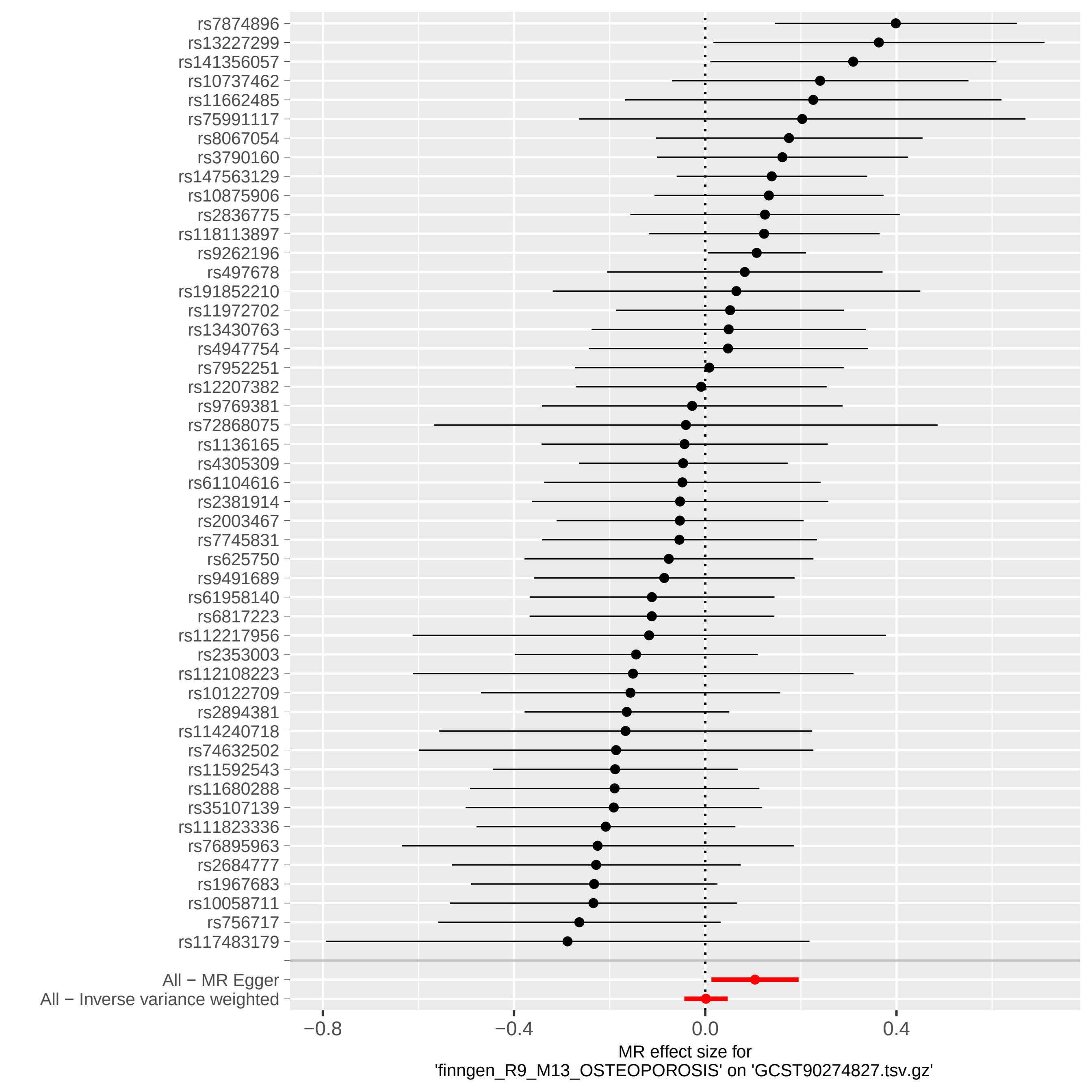
rs7874896
rs13227299
rs141356057
rs10737462
rs11662485
rs75991117
rs8067054
rs3790160
rs147563129
rs10875906
rs2836775
rs118113897
rs9262196
rs497678
rs191852210
rs11972702
rs13430763
rs4947754
rs7952251
rs12207382
rs9769381
rs72868075
rs1136165
rs4305309
rs61104616
rs2381914
rs2003467
rs7745831
rs625750
rs9491689
rs61958140
rs6817223
rs112217956
rs2353003
rs112108223
rs10122709
rs2894381
rs114240718
rs74632502
rs11592543
rs11680288
rs35107139
rs111823336
rs76895963
rs2684777
rs1967683
rs10058711
rs756717
rs117483179
All − MR Egger
All − Inverse variance weighted
−0.8
−0.4
0.0
0.4
MR effect size for
'finngen_R9_M13_OSTEOPOROSIS' on 'GCST90274827.tsv.gz'
